# Several Affinity Tags Commonly Used in Chromatographic Purification

**DOI:** 10.1155/2013/581093

**Published:** 2013-12-26

**Authors:** Xinyu Zhao, Guoshun Li, Shufang Liang

**Affiliations:** State Key Laboratory of Biotherapy, West China Hospital, Sichuan University, No. 17, Third Section of Renmin South Road, Chengdu 610041, China

## Abstract

Affinity tags have become powerful tools from basic biological research to structural and functional proteomics. They were widely used to facilitate the purification and detection of proteins of interest, as well as the separation of protein complexes. Here, we mainly discuss the benefits and drawbacks of several affinity or epitope tags frequently used, including hexahistidine tag, FLAG tag, Strep II tag, streptavidin-binding peptide (SBP) tag, calmodulin-binding peptide (CBP), glutathione S-transferase (GST), maltose-binding protein (MBP), S-tag, HA tag, and c-Myc tag. In some cases, a large-size affinity tag, such as GST or MBP, can significantly impact on the structure and biological activity of the fusion partner protein. So it is usually necessary to excise the tag by protease. The most commonly used endopeptidases are enterokinase, factor Xa, thrombin, tobacco etch virus, and human rhinovirus 3C protease. The proteolysis features of these proteases are described in order to provide a general guidance on the proteolytic removal of the affinity tags.

## 1. Introduction

The expression and purification of recombinant proteins have become increasingly common for characterizing structure and function of proteins in recent years. There is a need to purify the protein of interest to obtain enough concentration with high purity before its function, structure and interactions with other proteins can be studied. Various methods have been used to enrich proteins of interest from crude biological extracts. The most effective method is affinity purification, whereby the protein of interest is enriched by virtue of its specific binding properties to an immobilized ligand function in a fashion similar to that of antibody-antigen interactions. Affinity or epitope tags are peptide sequences, which are extremely powerful tools and often appended to the target protein of interest. Initially affinity tags have been devised to purify recombinant proteins, but now they are also used in western blot, immunohistochemistry (IHC), immunoprecipitation (IP), flow cytometry (FCM), protein localization, and so forth. However, each tag has its own distinct advantages and disadvantages [1, 2], which are important to consider before the final selection of tag to be used. This depends on the application and the requirement for specificity, solubility, binding, and elution conditions.

Generally, tags used to improve the production of recombinant proteins can be roughly divided into purification and solubility tags [3]. Affinity tags include enzymes, protein domains, or small polypeptides and most of which bind with high specificity to a range of substrates, such as carbohydrates, small biomolecules, metal chelates, antibodies, and so forth, to allow rapid and efficient purification of proteins. While the solubility tags enhance the proper folding and solubility of a protein, they are frequently used in tandem with an affinity tag to aid purification. In this context, we summarize the features and applications of several common affinity tags which are available for prokaryotic and eukaryotic protein expression systems (Table 1).

## 2. Affinity Tags and Their Features

**Figure 1:**
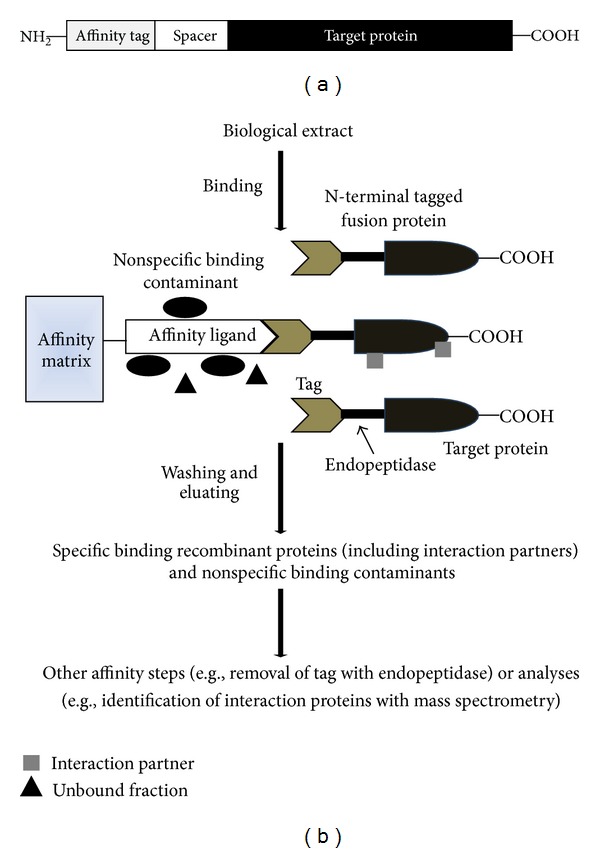
(a) Schematic illustration of the N-terminal tagged fusion protein. The spacer represents an endopeptidase cleavage sequence and/or solubility and folding enhancers. (b) Principle of fusion protein affinity purification and removal of the tag (only for N-terminal tagging). The interaction proteins will be copurified with the tagged fusion protein under native conditions.

Expression of recombinant protein in *Escherichia coli* (*E. coli*) or mammalian cells as a fusion protein with neighboring affinity tag is one of the most popular methods for purification of protein or protein complex. Affinity tags are artificial polypeptides which were usually grafted either onto the N- or C-terminus of a target protein through inserting the cDNA sequence which encoded the tag peptide into a matching open reading frame of the target protein (Figure 1). In addition to facilitating the purification of recombinant proteins, affinity tags can also enhance the yield, solubility, and even folding of the target partners [2, 4]. The small-size tags (e.g., 6× His, FLAG, Strep II, and CBP) have the benefits of minimizing the effect on the structure, activity, and characteristics of the recombinant protein, and therefore usually there is no need to remove. Large-size tags, including MBP and GST, have positive influences on protein solubility and expression efficiency, but the immunogenicity and the more consumption of cell metabolic energy in overexpressing cells are the major drawbacks compared with the small-size tags.

The hexahistidine tag (6× His-tag) is the most frequently used affinity tag for protein enrichment. His-tagged proteins can be purified easily by the chelated metal ions as affinity ligands. The basis for affinity purification is known as immobilized metal affinity chromatography (IMAC) [5]. His-tag can bind best to IMAC resin in near-neutral buffer conditions (physiologic pH and ionic strength), and thus the fusion proteins can be eluted with binding buffer containing certain concentrations of imidazole. If possible, the elution is also accomplished with low pH (e.g., 0.1 M glycine-HCl, pH 2.5) or an excess of strong chelator (e.g., EDTA). The 6× His-tag has several merits, including a smaller size, absence of electric charge, low levels of toxicity, and immunogenicity [3]. His-tagging provides good yields of fusion protein from inexpensive, high capacity resins with moderate purity from *E. coli* extracts but relatively poor purification from mammalian cell extracts [5]. For example, we expressed a recombinant protein FAM92A1-289 fused with 6× His-tag in *E. coli* and purified it using Ni^2+^-charged affinity resin for further function studies [6]. In the prokaryotic expression system, most of the recombinant His-tagged protein exists in form of inclusion body, and finally about 4 mg of FAM92A1-289 protein was obtained with high purity from 1 L of *E. coli* culture [6].

The maltose-binding protein (MBP) was one of the affinity tags to be used for the purposes of overcoming problems associated with the expression and purification of fusion proteins [7]. Generally, recombinant proteins tagged with MBP can alleviate toxicity and improve expression level and protein solubility [8–10]. MBP tagging may produce a higher percentage of recombinant protein than that the polyhistidine tag does [11, 12]. However, the disadvantage of MBP is the size and immunogenicity of the affinity tag, which complicates any downstream application. The purification of MBP-tagged proteins is achieved by conventional amylose resin-based chromatography. The elution of the MBP-fused proteins is at neutral pH using mild maltose-containing buffer conditions [13]. MBP tag is effective when placed on the N-terminal or C-terminal end of target proteins. However, because the large size of this MBP tag puts a heavy metabolic load on the host cell, the target protein remains insoluble or is prone to aggregation when the MBP tag is removed [14]. In addition, recently a novel SUMO fusion tag appears to enhance protein expression and solubility in prokaryotes and eukaryotes [14, 15].

The glutathione *S*-transferase (GST) tag is another well-established affinity tag based on the strong affinity of GST for immobilized glutathione [16]. The GST tag is best suitable for use in prokaryotic expression because GSTs are a family of multifunctional cytosolic proteins that are present in eukaryotic organisms but generally not found in bacteria [17]. Similar to the MBP tag, GST tag has long been used to increase the solubility of fusion proteins in *E. coli* [18]. GST-tagged proteins are captured by immobilized glutathione and then are eluted under mild, nondenaturing conditions using reduced glutathione [19].

The Strep-tag is an octapeptide that binds to streptavidin [20, 21]. Streptavidin was also optimized to increase peptide-binding capacity, which resulted in the development of Strep-Tactin. The streptavidin derivative, namely, Strep-Tactin, leads to a higher affinity for Strep II tag [22–24]. Strep II tag does not interfere with folding or bioactivity and does not induce protein aggregation either. Strep fusion proteins can be captured by Strep-Tactin ligand immobilized on the base matrix and purified in one step from crude cell extracts under physiological conditions, and thus the tag is especially applied to the generation of functional proteins or protein complexes [25]. At the same time, Strep II tag may provide an acceptable compromise of excellent purification with pure yields at a moderate cost [6]. In addition, the 38-amino acid streptavidin-binding peptide (SBP) tag was developed, which binds to streptavidin more tightly than the Step-tag II and the native tag [26]. The Strep-tagged or SBP-fused proteins can be dissociated from the ligand covalently attached to agarose resin by elution buffer with biotin or desthiobiotin [27, 28].

The calmodulin-binding peptide (CBP) tag was invented to purification of recombinant protein from bacteria based on high affinity for calmodulin with nanomolar affinity at physiological conditions in the presence of calcium [29]. The CBP tag is derived from the C-terminal fragment of human muscle myosin light-chain kinase and thus is not recommended for purification of fusion proteins in eukaryotic cells because endogenous proteins can interfere with calmodulin in a calcium-dependent manner [30]. Similar to the hexahistidine and the Strep II tags, CBP tag has a negligible impact on the biological activity or physical characteristics of the target partner. CBP-fused proteins are eluted with a strip of calcium from the environment under very moderate buffer conditions (e.g., 2 mM EGTA, pH 8.0) [31].

The chitin-binding domain (CBD) from Bacillus circulans consists of 51 amino acids, which is commonly used as tags for affinity purification of recombinant proteins in combination with self-splicing inteins in bacterial systems [32]. Following affinity selection of the fusion protein on a chitin matrix, the intein undergoes specific cleavage by a thiol reagent or pH and temperature shift which releases the target protein from the chitin-bound tag [3].

The FLAG tag is a hydrophilic octapeptide epitope tag that was introduced to purify fusion proteins [33]. It is likely to be located on the surface of a fusion protein because of its hydrophilic nature and therefore is more likely to be accessible to antibodies. FLAG tag binds to several specific anti-FLAG monoclonal antibodies such as M1, M2, and M5 with different recognition and binding characteristics [34, 35]. FLAG fusion proteins can be recognized by monoclonal antibody with calcium-dependent (e.g., M2) or calcium-independent manner [32]. In particular, the tag appended to the N-terminus of the fusion protein is necessary for the immunoaffinity purification with M1 monoclonal antibody, while M2 is position-insensitive. The elution of the FLAG-tagged proteins is performed with FLAG peptide (e.g., 3× FLAG peptide) or low pH glycine buffer (e.g., 0.1 M glycine, pH3.5) [36].

The S-tag system is based on the specific binding between 15-amino acids S-tag and S-protein, both of which are derived from pancreatic ribonuclease A (RNase A). Any protein fused with the S-tag can be conveniently purified, detected, and even quantified [37–39]. However, the elution of S-tagged proteins is performed under highly stringent condition in the presence of 3 M NaSCN, 3 M MgCl_2_, or 0.2 M citrate (pH 2).

Besides the affinity tags described above, other polypeptides such as the HA tag and the c-Myc tag are well characterized and highly immunoreactive tags which are generally used for the separation of tagged proteins from cell culture supernatants and cell lysate under neutral pH conditions and thus are handy tools for coimmunoprecipitation (co-IP) but are also easily detected via western blot. Moreover, they are small and thus unlikely to interfere with the bioactivity and function of the fusion partner proteins. HA tag comes from human influenza hemagglutinin (HA) corresponding to amino acids 98–106 and is a strong immunoreactive epitope making it popular to isolate, purify, detect, and track the protein of interest [40, 41]. The recombinant HA-tagged proteins can be separated by highly specific anti-HA monoclonal antibody that is covalently immobilized on resin. The HA-tagged proteins can be eluted by mild elution approach with HA epitope at 1 mg/mL in TBS. On the other hand, three chemical elution options are available: 0.1 M glycine (pH 2–2.8), 3 M NaSCN, or 50 mM NaOH.

The c-Myc tag originates from the *c-myc* gene product. The recombinant protein tagged with c-Myc tag can be recognized by a well-known high-affinity 9E10 antibody [42]. Though it can be added to the C-terminus or N-terminus of a protein, it is not recommended to append the c-Myc tag directly behind the signal peptide of a secretory protein because the tag can interfere with translocation into the secretory pathway. In any case, the c-Myc tag can be used in many different assays such as subcellular localization studies by immunofluorescence or detection by western blot. Under native conditions, the elution of c-Myc-tagged proteins can be achieved by the addition of the c-Myc tag peptide (0.5 mg/mL in PBS) which competes with the recombinant proteins.

## 3. Combinatorial Tagging Strategy and the Studies of Protein Interacting Partners

Protein complexes and protein-protein interactions constitute the functional bases of the life activities within the cell. Many tag combinations have been developed since the tandem affinity purification (TAP) technique appeared at late 1990s [43]. TAP-tagging, which employs two sequential affinity purification steps, can significantly reduce the chance of contaminants retained in the eluate. Double-affinity tag is an efficient approach for the purification protein complexes under native conditions [44, 45]. As a powerful tool to separate interacting protein complex, TAP-tagging strategy is widely used in the studies of protein interaction networks. The combination of TAP technique with mass spectrometry (MS) has been widely adopted as a highly efficient method to identify and characterize the components of protein complexes [46–49]. For example, we developed a TAP tag system containing a FLAG and a CBP tag to purify binding partners with a bait protein 14-3-3*ε* in mammalian cells, and a new interacting protein HSP70 was identified by two steps of affinity purification [48].

Various combinations of different tags have been reported so far, such as His and FLAG, His and Strep II, FLAG and Strep II, and so on [50, 51]. Fortunately, many TAP expression vectors are commercially available today. Altogether, the adoption of tag in TAP strategy needs to carefully determine according to the advantages and disadvantages of various tags and the characteristics of a target protein. To choose an effective combination, it is normally necessary to consider the abilities of the tags to improve the yield, enhance the solubility, and facilitate the purification of their fusion partners. Additionally, if affinity tags have the potential to interfere with structural or functional studies, the fused tag must be removed from the bait protein as follows.

## 4. Removal of Affinity Tags

The use of affinity tag for the purification of proteins in both prokaryotic and eukaryotic expression systems is a well-accepted method. In theory, it cannot be excluded that affinity tags, especially those with large size, may have the potential to interfere with the structure and function of the proteins. If this circumstance happens, measures should be made for removing them. Any affinity tag, whether small or large, can be easily removed by introducing a specific protease recognition sequence between the tag and target protein (Figure 1(b)). The most frequently used endopeptidases are enterokinase, factor Xa, thrombin, tobacco etch virus (TEV), and human rhinovirus 3C protease. The advantages and disadvantages of these endopeptidases were thoroughly discussed in the previous works of the literature [1, 52–54]. Moreover, other endopeptidases (e.g., PreScission and Sortase A) and exopeptidases (e.g., DAPase, *Aeromonas* aminopeptidase, aminopeptidase M, and carboxypeptidase A and B) were described exhaustively for the removal of affinity tags from recombinant proteins [1, 52].

The small-size tags such as 6× His, FLAG, Strep II, and CBP usually do not need to be removed for downstream applications following purification. Enterokinase possesses trypsin-like activity and specifically cleaves after lysine residue at a canonical recognition sequence (DDDDK↓) [55]. Enterokinase may sometimes cleave at other basic residues, depending on the conformation of the protein substrate [56–60]. In addition, the cleavage efficiency of enterokinase is closely associated with the amino acid residue after downstream of the recognition site [61, 62]. It will not cleave at the recognition site if the recognition sequence is followed by proline. However, the addition of urea (1–4 M) can greatly improve enterokinase cleavage specificity and reduce adventitious cleavage [63]. In particular, FLAG tag (DYKDDDDK) contains the enterokinase cleavage site (underlined) which allows the removal of affinity tag after purification.

As the enterokinase, both thrombin and factor Xa are trypsin-like serine proteases which will cleave peptide bonds on the carboxyl side of a basic amino acid residue. Factor Xa cleaves after the arginine residue in its preferred cleavage site (I-E/D-G-R↓) and occasionally cleaves at other sites [64–66]. Similar to the enterokinase, factor Xa will not cleave at a site followed by proline or arginine. For the most common recognition sequence (LVPR↓G) or (LVPR↓GS), thrombin will selectively cleave after arginine residue [54]. Thus, unlike enterokinase and factor Xa, thrombin cleavage may result in the retention of one or two amino-terminal amino acid residues in protein of interest.

The TEV protease is a highly site-specific cysteine endoprotease. Its optimum recognition sequence is E-N-L-Y-F-Q↓-G/S and the cleavage occurs between the glutamine and glycine/serine residues [67].

Sortase A is a prokaryotic thiol transpeptidase that can hydrolyze the fusion proteins by recognizing a carboxyl-terminal sorting signal sequence (LPET↓G) and cleaving the threonine-glycine peptide bond [68, 69]. The cleavage activity of the enzyme can be stimulated by calcium ions [70], and an additional affinity step is needed for on column tag removal such as using immobilized sortase A [71].

PreScission protease is a GST tagged human rhinovirus (HRV) type 14 3C protease and specifically recognizes the amino acid sequence LEVLFQ↓GP or its subset of sequences which include the core amino acid sequence (underlined), cleaving between the glutamine and glycine residues [72, 73]. The recombinant enzyme is specifically designed to facilitate removal of the protease by allowing simultaneous protease immobilization and cleavage of GST affinity tag.

Besides the tag cleavage methods described above, removal of the tag without using a protease is also feasible by introducing a protein element with self-splicing capacity into a variety of tag-based purification systems. These elements are naturally self-splicing proteins called inteins that can excise themselves from the parent protein [74, 75]. Inteins can be designed at N- or C-terminal splice junctions to obtain self-cleaving inteins, which can then be used to achieve self-cleaving of various affinity tags. The specific applications have been comprehensively reviewed by other authors [76–78].

## 5. Conclusions

Affinity purification is an approach of isolating biomoleculars from cell extracts based on a highly specific interaction as that between antigens and antibodies, as well as receptors and ligands. Affinity tag is absolutely necessary for many applications in life sciences, including the purification of protein of interest. Although each tag has its specific advantages and disadvantages in purification efficiency, the versatile affinity purification systems with different affinity tags are powerful to isolate recombinant protein and protein complexes. To sum up, there is no universal affinity purification system for exploring different bait protein and its binding partners at present. So far, it is feasible to generate multiple purified human proteins or protein domains for affinity proteomics and large-scale structural genomics studies by now [79].

## Figures and Tables

**Table 1:** Common widely used affinity tags for purification of recombinant proteins.

Affinity tag	Length (aa)	Size (kDa)	Matrix
Hexahistidine (6x His)	6 (generally)	0.84	Metal ions (Ni^2+^, Co^2+^, Cu^2+^, Zn^2+^, Fe^3+^)
Glutathione S-transferase (GST)	211	26	Glutathione
FLAG	8	1.01	Anti-FLAG mAb
Streptavidin-binding peptide (SBP)	38	4.3	Streptavidin
Strep II	8	1.06	Strep-Tactin (modified streptavidin)
Maltose-binding protein (MBP)	396	42	Amylose
Calmodulin-binding peptide (CBP)	26	2.96	Calmodulin
Chitin-binding domain (CBD)	51	5.59	Chitin
S	15	1.75	S-protein of RNase A
HA	9	1.1	Anti-HA epitope mAb
c-Myc	11	1.2	Anti-Myc epitope mAb
